# New Rising Infection: Human Herpesvirus 6 Is Frequent in Myeloma Patients Undergoing Autologous Stem Cell Transplantation after Induction Therapy with Bortezomib

**DOI:** 10.1155/2012/409765

**Published:** 2012-11-29

**Authors:** Netanel Horowitz, Ilana Oren, Noa Lavi, Tsila Zuckerman, Noam Benyamini, Zipi Kra-Oz, Viki Held, Irit Avivi

**Affiliations:** ^1^Department of Hematology and Bone Marrow Transplantation, Rambam Health Care Campus, P.O. Box 9602, Haifa 31096, Israel; ^2^Unit of Infectious Diseases, Rambam Health Care Campus, P.O. Box 9602, Haifa 31096, Israel; ^3^Bruce Rappaport Faculty of Medicine, Technion – Israel Institute of Technology, P.O. Box 9602, Haifa 31096, Israel; ^4^Virology Laboratory, Rambam Health Care Campus, P.O. Box 9602, Haifa 31096, Israel

## Abstract

Herpesvirus 6 (HHV-6) infection is a common complication during immunosuppression. Its significance for multiple myeloma (MM) patients undergoing autologous stem cell transplantation (ASCT) after treatment with novel agents affecting immune system remains undetermined. Data on 62 consecutive MM patients receiving bortezomib-dexamethasone (VD) (*n* = 41; 66%) or thalidomide-dexamethasone (TD) (*n* = 21, 34%) induction, together with melphalan 200 mg/m^2^ autograft between 01.2005 and 09.2010, were reviewed. HHV-6 reactivation was diagnosed in patients experiencing postengraftment unexplained fever (PEUF) in the presence of any level of HHHV-6 DNA in blood. There were no statistically significant differences in patient characteristics between the groups, excluding dexamethasone dosage, which was significantly higher in patients receiving TD. Eight patients in TD and 18 in VD cohorts underwent viral screening for PEUF. HHV-6 reactivation was diagnosed in 10 patients of the entire series (16%), accounting for 35% of those screened; its incidence was 19.5% (*n* = 8) in the VD group versus 9.5% (*n* = 2) in the TD group. All patients recovered without sequelae. In conclusion, HHV-6 reactivation is relatively common after ASCT, accounting for at least a third of PEUF episodes. Further studies are warranted to investigate whether bortezomib has an impact on HHV-6 reactivation development.

## 1. Introduction

Human herpesvirus 6 (HHV-6) is highly prevalent in humans, infecting almost all children during their early childhood [1–4]. Similar to other herpesviruses, it tends to remain dormant in the host tissues, but may reactivate in the presence of immune suppression, resulting in a febrile illness, often accompanied with skin eruption, encephalitis, or pneumonia [5–11]. Immune dysfunction, existing following allogeneic stem cell transplantation (Allo SCT), induced by either immunosuppressive drugs or the development of graft-versus-host disease (GvHD) appears to result in a significant risk of HHV-6 reactivation [12], approaching 33–48% [13–16]. 

Unlike Allo SCT, autologous stem cell transplantation (ASCT), being associated with mild transient immunodeficiency, has been traditionally considered a less probable cause of HHV-6 reactivation. However, studies exploring this risk in heterogeneous groups of autografted patients reported a similarly high risk for HHV-6 infection [16], suggesting the malignancy itself, and/or the treatment applied preautograft, to contribute to transplant-associated immune impairment.

Induction therapy for myeloma has changed dramatically over the last few years and the traditional VAD (vincristine, doxorubicin, dexamethasone) has been substituted with thalidomide, bortezomib, and lenalidomide-based regimens [17, 18]. Apart from their well-recognized tumoricidal activity, these novel agents are known to modulate myeloma cell microenvironment and the immune system. Lenalidomide and thalidomide act as immunomodulatory drugs, inducing activation of cytotoxic T lymphocytes, and natural killer (NK) cells [19, 20], whereas bortezomib-based induction has been recently suggested to cause a transient decrease in CD8 and NK cell counts [21] and function, selective depletion of TH_1_ cells [22] and dendritic cell (DC) dysfunction [23, 24]. 

This leads to an increased incidence of herpes Zoster infection in patients receiving bortezomib, which is reported to approach 13% [25]. 

The incidence of HHV-6 infection has not yet been studied in a large homogenous group of autografted MM patients. Furthermore, a potential adverse effect of pretransplant administration of novel biological agents on transplant-related HHV-6 infection has not been explored.

The current study was designed to assess the incidence, clinical significance, and risk factors for HHV-6 reactivation in a large cohort of myeloma patients, consecutively treated with novel agents and ASCT. 

## 2. Patients and Methods

The study was approved by the Institutional Review Board (IRB) of the Rambam Health Care Campus (approval no. 0380-11RMBG). 

The departmental transplant database was searched for all myeloma patients, aged 18 years or older, who underwent autologous stem cell transplantation after receiving a thalidomide or bortezomib-based therapy, completed within less than 2 months to prior transplant. Patients who received VT or TD as their second line pretransplant therapy were not included, unless they completed first therapeutic regimen, at least 6 months prior to the initiation of TD/VD. Patients receiving a second ASCT, performed in a tandem setting or at disease progression, were not included in this study. Data on induction therapy and transplant-related complications (particularly, infectious, neurological, and respiratory) were obtained from original computerized medical files. 

### 2.1. Treatment Protocols for Multiple Myeloma

Pretransplant treatment protocols included in the current study were VD (intravenous bortezomib 1.3 mg/m^2^ on days 1, 4, 8, 11, and dexamethasone 40 mg on the days of bortezomib administration) and TD (p.o. thalidomide 100–200 mg daily, administered together with dexamethasone).

The transplant conditioning regimen was melphalan 200 mg/m^2^, administered over 1 day. All transplanted patients received anti-herpes-zoster prophylaxis with acyclovir 200 mg four times per day, started on day +1 after transplant and continuing up to 90 days.

### 2.2. Virology Screening Protocol

According to the department protocol, patients with persistent fever (temperature >38°C for ≥3 days), occurring after neutrophil engraftment (>500) or beyond day 16 after transplant, in whom detailed investigation for a causative pathogen (multiple blood cultures, polymerase chain reaction (PCR) for aspergillus, serologic test for galactomannan, and chest CT scan) failed to detect bacterial or fungal infective cause, underwent a molecular investigation for viral infection, including PCR tests of peripheral blood (PB) for cytomegalovirus (CMV), Epstein-Barr virus (EBV), adenovirus, and HHV-6. Patients exhibiting lower or upper respiratory symptoms were also screened for respiratory viruses, using PCRs for influenza (A, B, H1N1), parainfluenza viruses types 1, 2, 3, RSV, and adenovirus, examined in respiratory secretions. Notably, criteria for performing virology screen remained unchanged during the study period.

PCR for HHV-6 was repeated only if patient's symptoms (including fever) had not resolved within one week from HHV-6 diagnosis.

### 2.3. Diagnosis of HHV-6 Reactivation

Diagnosis of HHV-6 reactivation was made in the presence of any level of HHV-6 DNA in blood [12] and otherwise unexplained fever after ruling out other infectious pathogens, as detailed above. High-level HHV-6 reactivation was defined as >1000 HHV-6 DNA copies/Ml plasma [12]. HHV-6 disease was diagnosed in the presence of HHV-6 related complications such as encephalitis or pneumonia [12].

Notably, the PCR test, described below, was shown to be highly sensitive (100%) and highly specific, with no false positive events, attributed to concurrent bacterial and viral infections [26].

### 2.4. DNA Extraction

DNA was extracted from patient's whole blood samples (200 *μ*L per sample), using the Magna Pure LC apparatus and Magna Pure LC total Nucleic Acid Isolation Kit Reagents (Roche Diagnostics, Germany). For manual extraction the QIAamp DNA blood mini kit was used, according to the manufacturer's instructions (QIAgen LTd., Crawly, England, UK). Up to the year 2008, HHV-6 DNA was detected using nested PCR (nPCR), which was then replaced with TaqMan real time PCR. 

### 2.5. Nested PCR

DNA was amplified by conventional nested PCR [27], in which the primers were designed to target the HHV-6 13R gene (the outer forward: 5′ AAG-CTT-GCA-CAA-TGC-CAA-AAA-AAC-AG-3′; Outer reverse: 5′ CTC-GAG-TAT-GCC-GAG-ACC-CCT-AAT-C-3′; Inner forward: 5′ TCC-ATT-ATT-TTG-GCC-GCA-TTC-GT-3; the Inner reverse: 5′ TGT-TAG-GAT-ATA-CCG-ATG-TGC-GT-3′). These primers present a genomic region showing high homology (95%) between HHV-6 A and B subtypes.

nPCR reaction was performed in 25 *μ*L volume using ReddyMix PCR master mix (Thermo scientific, UK). Five microliters (*μ*L) of the extracted DNA were added to the first PCR reaction and 2 *μ*L of the first round product were added to the nested reaction. Amplification was performed by 30 cycles of denaturation at 95°C for 60 seconds, annealing at 55°C for 60 seconds, and elongation at 72°C for 90 seconds. PCR products were detected by electrophoresis in 2% agarose gel stained with ethidium bromide.

### 2.6. TaqMan Real Time PCR

The primers and probe were targeted—U6 gene. (Forward primer: 5′ AAAATTTCTCACGCCGGTATTC 3′; reverse primer: 5′ CCTGCAGACCGTTCGTCAA 3′; probe—6-FAM-TCGGTCGACTGCCCGCTACCA-BHQ). PCR reaction was performed in a total volume of 25 *μ*L containing Absolute Blue QPCR mix (Thermo scientific UK) in the presence of 5 *μ*L target DNA, 300 nM of each primer, and 200 nM of the probe. PCR was performed on the Corbet Research platform under the following conditions: 15 min at 95°C, and 45 cycles of 15 seconds at 95°C and 60 seconds at 60°C. For quantitative results analysis, a standard curve was constructed using quantified HHV-6 DNA (Advanced Biotechnology Industry). The results were reported as the number of HHV-6 genome copies per 1 mL of blood. The lowest detection level of the test is 250 genomic copies per milliliter. 

## 3. Statistical Analysis

Analysis was performed using SPSS 18.0 software. As data were not normally distributed according to Kolmogorov-Smirnov test, quantitative variables were analyzed by Mann-Whitney *U* test. Categorical variables were analyzed by Fisher Exact test. *P* < 0.05 was considered significant.

## 4. Results

### 4.1. Characteristics of the Entire Patient Series

Sixty-two consecutive patients, 21 (33%) who received pretransplant TD and 41 (66%) who had VD, were analyzed. The median age at SCT for the whole series was 56.5 years (35–67 years). Characteristics of the patient group as a whole and depending on pretransplant induction regimen are presented in Table 1. No statistically significant differences were revealed in the characteristics of patients treated with VD versus TD, except for a longer time from diagnosis to SCT and a higher cumulative dose of steroids in those treated with TD (10 versus 8 months, *P* = 0.024, and 1000 versus 640 mg, *P* = 0.024, Table 1).

### 4.2. Characteristics of Patients Selected for Viral Screening

**Figure 1:**
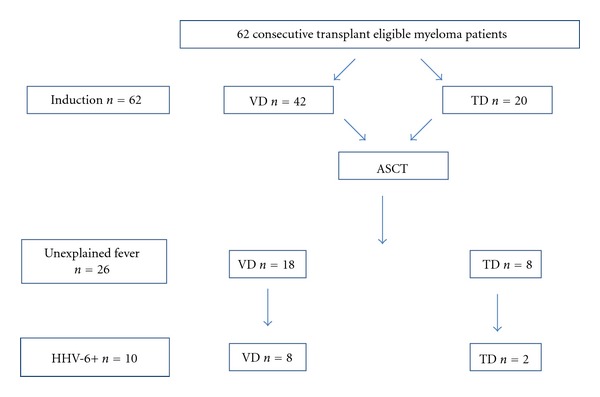
Cohort diagram.

Twenty-six patients, 8 treated with TD and 18 treated with VD (*P* = n.s.), who exhibited postengraftment unexplained fever, underwent molecular blood tests for viral infections (Figure 1). Characteristics of patients selected for viral screening after treatment with TD (*n* = 8) versus VD (18) are shown in Table 2. There were no statistically significant differences between these 2 cohorts apart from a higher steroid dosage in patients receiving TD (880 mg versus 640 mg, *P* = 0.022). 

### 4.3. Incidence of HHV-6 Reactivation and Characteristics of Infected Patients

HHV-6 reactivation was revealed in ten patients (median age 57 years; range 49–67 years), within 14 to 26 days after transplant (median 17 days). 

The incidence of reactivation in the whole cohort approached 16%: 19.5% (8/41) in the VD cohort, compared to 9.5% (2/21) in the TD group (*P* = n.s.). While a similar proportion of patients underwent screening in both treatment groups (43% of VDs versus 38% of TDs), the incidence of HHV-6 reactivation in the screened “VD subjects” approached 44% (8/18) versus 25% (2/8) in their “TD” counterparts (*P* = n.s.) (Figure 1). 

Notably, the cumulative steroid dose in patients diagnosed with HHV-6 reactivation was higher than recorded in screened HHV-6 negative subjects (880 mg versus 640 mg, *P* = 0.038) and was remarkably elevated, approaching 1230 mg, in those diagnosed with high-level HHV-6 reactivation (*n* = 3). 

All patients diagnosed with reactivation of HHV-6 presented with high, unexplained fever. 

Causes for fever in patients undergoing a virology screen, in whom HHV-6 was found to be negative, were considered as Hickman-related infection (resolving shortly after removal of Hickman catheter, *n* = 7), drug-related (resolving immediately after drug cessation; *n* = 1), or undetermined (*n* = 8), with no significant differences in distribution of these causes among patients treated with TD versus VD. 

Six of the 10 patients (60%) diagnosed with HHV-6 reactivation, were actually defined as having an HHV-6 disease, presenting with asymptomatic respiratory involvement, detected by chest CT scans, which demonstrated non-specific lung infiltrations. Notably, 3 of these 6 patients had a high level of HHV-6 reactivation. 

None of the patients diagnosed with HHV-6 reactivation developed delirium or any other clinically significant neurological manifestations, and none of the patients had a skin rash. 

The median time for neutrophil and platelet engraftment was similar for patients diagnosed with HHV6 infection versus the “negative” screened cohort, approaching 12 versus 14 days and 14 versus 15 days, respectively. 

As expected, all patients were severely lymphopenic at the time of reactivation (absolute lymphocyte count < 500/*μ*L). However, the median pretransplant lymphocyte count in patients diagnosed with HHV-6 reactivation did not statistically differ from that measured in the rest screened subjects (1045/*μ*L versus 730/*μ*L, resp.; *P* = 0.24). 

Infection-related symptoms self-resolved within one week after diagnosis in 9 patients, none of whom developed a concurrent opportunistic infection. 

One patient, experiencing prolonged fever (>1 week) since diagnosis of high-level HHV-6 reactivation (120,000 copies/*μ*L), underwent a repeated PCR test, still showing a significant number of viral copies (100,000 copies/*μ*L). PCR test for CMV was also positive, which is compatible with a concurrent CMV reactivation. Therefore, the patient was eventually treated with intravenous gancyclovir, resulting in resolution of clinical symptoms within 4 days. Three additional patients who experienced a spontaneous resolution of fever within less than one week but reported on a remarkable exhaustion, underwent a repeated PCR test (performed upon physician discretion), showing a low HHV-6 level in two (day 13 and 17, resp.), and clearance of HHV-6 in the third individual (day 14). 

A long-term evaluation, performed within a median followup of 494 days (range 14–2437) after autograft showed that 49 patients were alive, including 10 of the HHV-6 positive subjects (100%), 5 of the HHV-6 negative screened subjects (31%), and 8 of the nonscreened subjects (25%). None of the patients in the entire cohort had clinically significant long-term neurological sequels. 

### 4.4. Risk Factors for HHV-6 Infection

Univariate analysis of patients undergoing virology screen due to an unexplained fever following engraftment, found the exposure to a higher steroid dose to be the only statistically significant factor for HHV-6 infection (Table 3). Accumulative steroid dose in infected subjects approached 880 mg versus 640 mg in their “negative-screened” counterparts (*P* = 0.038). The incidence of HHV-6 infection appeared to be higher in subjects receiving VD versus TD (45% versus 25%), though the difference was not statistically significant. Notably, patients treated with VD received a statistically significantly lower dose of steroids (640 mg) relative to those treated with TD (880 mg) (Table 2). 

## 5. Discussion

The prevalence and significance of HHV-6 reactivation in patients undergoing ASCT have not been fully elucidated [16, 28, 29], which could be related either to a low incidence of this complication or poor reporting attributed to limited clinical significance. The largest series ever published, prospectively evaluating HHV-6 infection in autografted patients, observed a 47% risk for HHV-6 infection [16]. This study included 21 MM patients managed in the prenovel agent era, when treatment was based on chemotherapy rather than biological agents (e.g., thalidomide and bortezomib), which affected the immune microenvironment in addition to their direct antimalignant activity [30]. The relatively mild immunosuppression associated with ASCT might become more profound in patients whose pretransplant treatment already resulted in immunodeficiency. 

Bortezomib has been reported to induce T-cell inactivation [22], leading to an increased incidence of herpes zoster in patients treated with bortezomib-containing regimens [25, 31]. Interestingly, patients receiving thalidomide do not experience such risk, despite being exposed to equal doses of steroids. The expanding use of bortezomib for pretransplant induction therapy raised the question whether this novel agent also increases the risk of post-transplant HHV-6 reactivation. 

The current retrospective study explored the incidence and clinical significance of HHV-6 reactivation in 62 consecutive MM patients undergoing ASCT, after receiving induction therapy with either thalidomide or bortezomib. Notably, there were no statistically significant differences in characteristics of patients receiving VD versus TD, apart from a higher cumulative steroid dose in those receiving thalidomide. Twenty-six (42%) patients, 18 in the VD cohort (44%) and 8 in the TD group (38%), experienced a post-engraftment “unexplained” fever, hence, underwent a PCR virology screen. Ten patients (16.5%) were diagnosed with HHV-6 reactivation. This incidence appears to be lower than previously reported in autografted patients [16], reflecting either the lack of large homogenous historical series reliably estimating the incidence of HHV-6 reactivation in transplanted myeloma patients, or the retrospective nature of our study which selected patients for viral screen based on their symptoms. Thus, reporting was limited to clinically significant cases only, while missing asymptomatic infected subjects, those presenting with delayed engraftment as their sole complication (a presentation which is relatively rare), or those in whom fever resolved in less than 3 days. 

As mentioned earlier, previous studies looked at heterogeneous groups of autographed patients treated in the prenovel agent era; hence, their data are barely comparable with ours [16, 29]. 

All the ten patients diagnosed with HHV-6 reactivation, presented with high unexplained fever, in the absence of other opportunistic infections, except for one subject, who had concurrent reactivation of CMV, a phenomenon recently described by Zerr et al. [12]. Respiratory involvement, though clinically insignificant, was detected in 6 subjects, whereas none had clinical evidence of neurological involvement, emphasizing the relatively innocent course of HHV-6 reactivation in this population. These findings are in line with the low PCR level observed in most our patients, which was less than reported in the allograft setting [12]. Nonetheless, HHV-6 reactivation may result in a more aggressive course, characterized with a higher frequency of neurological complications [12, 32], and concurrent opportunistic infections, reflecting the deeper immunosuppressive milieu induced in the allogeneic setting and potentially resulting even in long-term neurological complications [12, 32]. 

However, the conclusion regarding the relatively innocent course of HHV-6 reactivation in autografted MM patients, previously treated with biological agents affecting immune function, should be considered with caution, given the retrospective nature of the study, the small number of evaluated patients, and the relatively short-term followup after transplant, which may interfere with proper evaluation of long-term infection-related sequels. 

Nevertheless, despite the mild clinical course of HHV-6 in this setting, HHV-6 reactivation appeared to be responsible for a significant number of “post-engraftment unexplained febrile episodes,” emphasizing the significance of searching for this pathogen as a potential cause for fever. 

Eight patients in the VD group and 2 in the TD cohort, accounting for 19.5% of the VD versus 9.5% of the TD series, developed HHV-6 reactivation. In a similar vein, the incidence of HHV-6 in tested VDs was higher than in tested TDs (44% versus 25%), despite screening a comparable proportion of patients in both treatment groups (44% in the VD versus 36% in the TD, *P* = n.s.). This difference, though not statistically significant due to the low number of patients analyzed, may suggest VD to be a risk factor for post-transplant HHV-6 reactivation. 

Treatment with bortezomib has been reported to be uniquely associated with an increased risk for varicella-zoster virus (VZV) infection [25]. The VZV incidence in VD-treated patients approached 13%, compared with 5% in their dexamethasone-treated counterparts (*P* = 0.0002) [25]. The mechanism of VZV reactivation has not yet been fully clarified. VZV-specific T cells appear to be necessary for suppressing VZV reactivation and preventing the VZV development [21, 33, 34]. Several studies suggested that bortezomib alters the number and function of specific lymphocyte subsets [21, 35]. Furthermore, it has been demonstrated that bortezomib impairs dendritic cell viability and function [23, 24], augmenting T-cell dysfunction. It is plausible that bortezomib also alters the function and/or interactions of key immune cells required for the suppression of HHV-6. 

Consistent with our results, exposure to steroids was reported to significantly increase the risk for herpetic diseases, particularly herpes zoster [36], inducing a marked immunosuppression which allows herpes reactivation. Indeed, a high level of HHV-6 reactivation was associated with a higher accumulative steroid dose.

It is noteworthy that the low incidence of HHV-6 reactivation observed in patients receiving thalidomide may reflect induction of immunomodulation, rather than immunodeficiency [37]. 

## 6. Conclusion

The current study, investigating the incidence and clinical significance of HHV6 reactivation in a large cohort of homogenous transplanted MM patients, suggests HHV-6 is a significant cause of unexplained post-engraftment fever. Although the infection is almost always self-resolving, its detection could exclude the necessity for additional investigations and help in the management of such patients. The findings may suggest bortezomib is a potential risk factor of HHV-6 reactivation development, emphasizing the need for further large prospective studies to confirm this observation.

## Figures and Tables

**Table 1:** Clinical characteristics of the group as a whole (*n* = 62).

	Whole group (*n* = 62)	VD cohort (*n* = 41)	TD cohort (*n* = 21)	*P*
Sex (male)	36 (58%)	25 (61%)	11 (52%)	n.s.
Median age, years (range)	56.5 (35–67)	58 (35–67)	56 (45–64)	n.s.
Median time from diagnosis to SCT, months (range)	9 (4–60)	8 (4–36)	10 (7–60)	0.024
Median accumulative steroid dosage, mg (range)	800 (320–4320)	640 (320–3680)	1000 (320–4320)	0.024

**Table 2:** Clinical characteristics of screened patients (*n* = 26) dependent on induction therapy.

	VD (*n* = 18)	TD (*n* = 8)	*P*
Sex (male)	9 (50%)	4 (50%)	
Median age, years (range)	55 (35–67)	54 (45–63)	n.s.
Disease status	CR; 2		
	PR: 1	PR: 5	
	VGPR: 9	VGPR: 2	
	Unknown: 6	Unknown: 1	
Median time from diagnosis to SCT, months (range)	7 (5–28)	9.5 (7–24)	n.s.
Median accumulative steroid dosage, mg (range)	640 (480–3680)	880 (320–4320)	0.022

**Table 3:** Risk factors for HHV6 reactivation after autologous SCT*.

	HHV-6 positive (*n* = 10)		HHV-6 negative (*n* = 16)		*P*
Age	57 (49–67)		53 (35–63)		n.s.
Male	7 (70%)		6 (37.5%)		n.s.
Female	3 (30%)		10 (62.5%)		n.s.
Median time from diagnosis to SCT, months (range)	8.5 (6–28)		7 (5–19)		n.s.

Induction regimen*	VD	TD	VD	TD	
	8 (44%)	2 (25%)	10 (54%)	8 (75%)	n.s.

Median accumulative steroid dosage, mg (range)	880 (640–1600)		640 (320–4320)		0.038
Pretransplant lymphocyte count (cells/*μ*L)	1045		730		0.24

*Calculation represents the proportion of HHV-6 infection in screened patients that were treated with the same induction regimen (VD or TD, resp.).
